# An Extremely Rare Nasopharyngeal Malignant Tumor: A Case Report

**DOI:** 10.7759/cureus.31444

**Published:** 2022-11-13

**Authors:** Paraskevi Karamitsou, Alexandros Poutoglidis, James Philip Skliris, Ioannis Matzarakis, Spyridon Gougousis

**Affiliations:** 1 Department of Otorhinolaryngology-Head and Neck Surgery, 'G. Papanikolaou' General Hospital, Thessaloniki, GRC; 2 Department of Pathology, 'G. Papanikolaou’ General Hospital, Thessaloniki, GRC; 3 Department of Pathology, 'G. Papanikolaou' General Hospital, Thessaloniki, GRC

**Keywords:** surgical excision, nasal obstruction, nasal septum, nasopharynx, nasopharyngeal papillary adenocarcinoma

## Abstract

Nasopharyngeal papillary adenocarcinoma (NPAC) is an extremely rare primary malignant tumor. There is only a limited number of cases of NPACs reported in the literature. The neoplasm presents as an exophytic mass with a papillary or polypoid appearance derived from the nasopharyngeal surface epithelium. It can potentially involve any part of the nasopharynx, but it most commonly involves the roof, the lateral, and the posterior wall. The prognosis is very good and no recurrences or metastases have been reported. Nasal obstruction is the most common manifestation. Surgical excision is considered the most appropriate treatment method. There are also reports of patients undergoing radiation therapy. However, its role in the treatment has not been clarified. The presence of this tumor in the nasal cavity could be easily underestimated, because of its appearance. As a result, an index of suspicion is necessary for a timely diagnostic and therapeutic intervention. We present a case of NPAC in a 26-year-old female treated in our hospital.

## Introduction

According to the fourth edition of the World Health Organization (WHO) classification of head and neck tumors, malignant epithelial tumors of the nasopharynx consist of nasopharyngeal carcinomas (NPCs), nasopharyngeal papillary adenocarcinomas (NPACs), and salivary gland carcinomas [1]. NPACs are primary malignant tumors derived from the nasopharyngeal surface epithelium and represent less than 1% of all nasopharyngeal malignancies [1]. They are invariably low-grade neoplasms and can potentially involve any part of the nasopharynx. Gender predilection has not been reported and NPACs occur over a wide age range (reported ages: 9-64 years) [1,2]. Nasal obstruction is the most common manifestation [1, 3]. Only a limited number of cases of NPACs has been reported in the literature because these neoplasms are extremely rare [4,5]. As clinical presentation could be easily underestimated, an index of suspicion is necessary for a timely diagnostic and therapeutic intervention. We present a case of NPAC in a 26-year-old female treated in our hospital.

## Case presentation

A 26-year-old, nonsmoker female presented to our ENT outpatient department with the chief complaint of a 2-month history of nasal obstruction. No other accompanying symptoms were reported. The patient’s medical history was otherwise normal.

**Figure 1:**
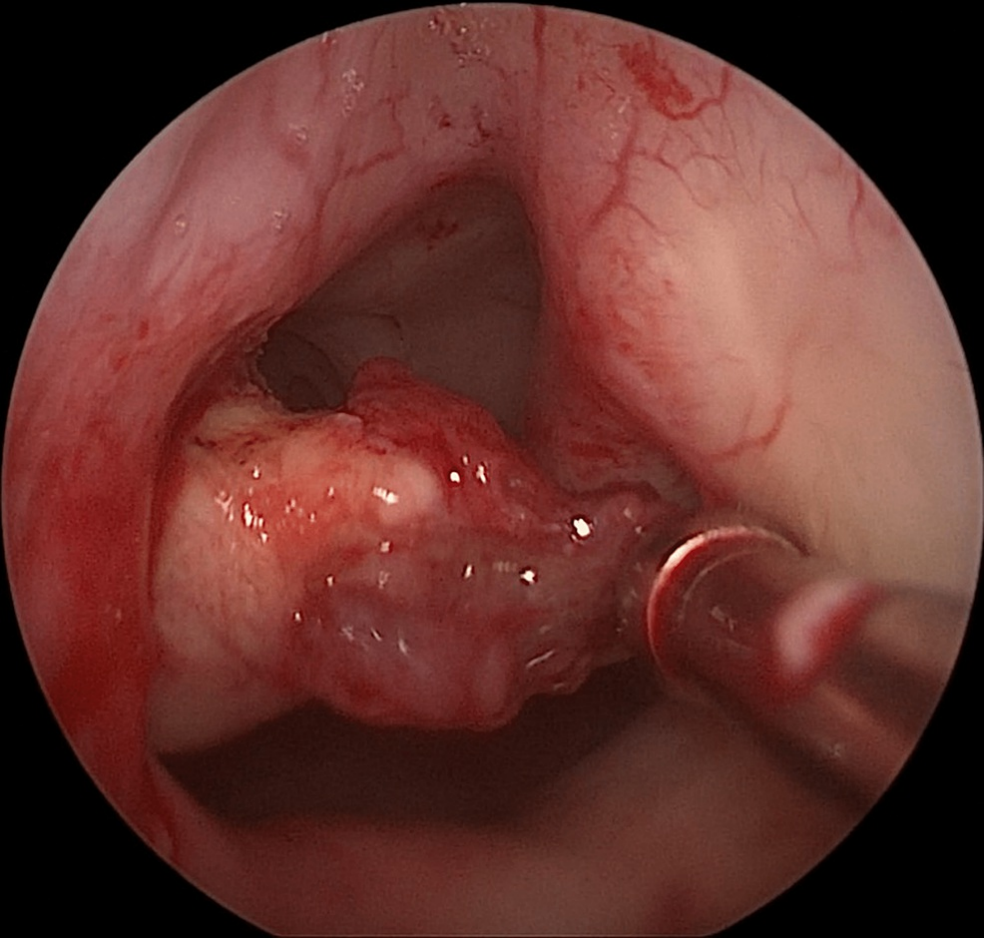
Endoscopic image Inflexible nasal endoscopy revealed an exophytic mass located on the left posterior third of the nasal septum

A thorough otolaryngological examination was performed. Inflexible nasal endoscopy revealed a soft, exophytic mass deriving with its stalk from the posterior third of the nasal septum. The mass occupied part of the posterior left half of the nasal cavity projecting into the nasopharynx, without adhesion to adjacent tissues (Figure 1). 

**Figure 2:**
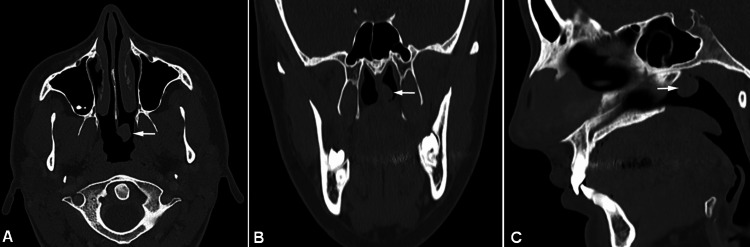
Sinus CT scan A polypoid mass (white arrow) (11x9x15mm) deriving from the nasal septum with no invasion of the surrounding structures. Mild enhancement of the mass after intravenous contrast agent administration. A. Axial, B, Coronal, C. Sagittal

Laboratory examinations demonstrated normal values. A sinus CT scan was performed showing a polypoid mass deriving from the nasal septum and measuring 11x9x15mm. The mass was mildly enhanced after intravenous contrast agent administration. No invasion of surrounding structures was noticed (Figure 2). 

**Figure 3:**
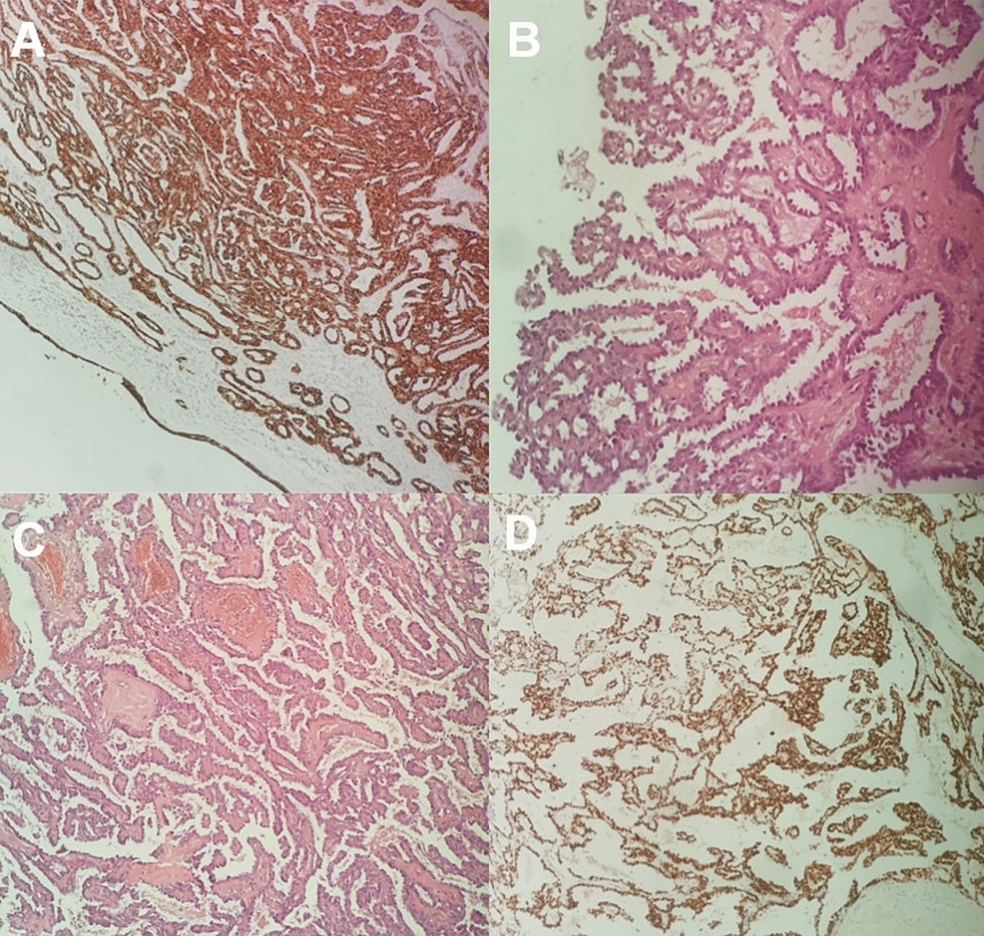
Histology A. The epithelial nature of the lesion was confirmed with pan-cytokeratin stain (Immunohistochemistry [IHC], 10X), B. Cuboidal neoplastic cells with minimal atypia line fibrovascular cores (H&E stain, 20X), C. Tumor components forming papillary and glandular formations (H&E, 10X), D. Cancerous cells displayed strong positivity for TTF-1 stain (IHC, 10X)

Subsequently, surgery was scheduled and the mass was completely removed via a transnasal endoscopic approach, under general anesthesia. The histology report indicated a low-grade NPAC (Figure 3). 

The tumor cells were arranged in glandular and papillary formations and their size was relatively uniform. No mitotic activity was reported. Immunohistochemistry showed pan cytokeratin (AE1/AE3), thyroid transcription factor-1 (TTF-1), and p53 positivity, while thyroglobulin expression was negative. Ki-67 proliferation index was ≤ 10%. The patient was discharged uneventfully from the hospital on the first postoperative day. 

Our hospital's multidisciplinary team (MDT) recommended a wider excision for oncologic purposes due to the tumor’s location and features. The MDT did not recommend adjuvant radiotherapy in absence of residual disease after the second intervention.

**Figure 4:**
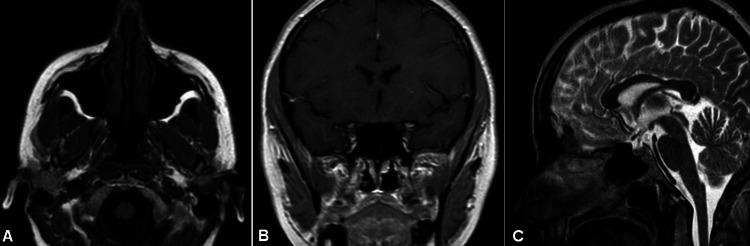
Sinus MRI Sinus MRI following endoscopic resection revealed normal findings. A. Axial (T1W1 sequence), B. Coronal (T1W1 sequence), C. Sagittal (T2W1 sequence)

A sinus MRI was also requested to evaluate the presence of residual disease. No abnormal findings were shown on MRI (Figure 4). 

A wider surgical excision was performed one week later via a transnasal endoscopic approach. Excision included part of the posterior third of the nasal septum with the overlying mucosa. The absence of residual disease and negative surgical margins were reported. The patient recovered well and was discharged on the second postoperative day. No adjuvant therapy was recommended except for close follow-up. Six months after the excision, the patient remains disease-free.

## Discussion

The most common malignant epithelial tumors of the nasopharynx are NPCs. NPC remains the diagnostic term of choice for all squamous cell carcinomas of the nasopharynx. Two other epithelial tumors appear in the nasopharynx. These are the NPACs and the salivary gland anlage tumors. A broad range of additional neoplasms can arise in the nasopharynx, including lymphoid, mesenchymal tissue, and neurogenic tumors [1].

NPAC may appear in any part of the nasopharynx, but it most commonly involves the roof, the lateral, and the posterior wall [4]. It presents as a soft, slow-growing exophytic mass with a papillary or polypoid appearance. Its size may vary from a few millimeters to approximately 3cm. Nasal obstruction is the most commonly presented symptom. Rhinorrhea, bleeding, otitis media, and hearing-related issues have also been reported [1,3].

On histopathological examination, NPACs are invariably low-grade tumors and composed of arborizing papillae and tubules lined by a single layer of bland columnar to cuboidal epithelial cells with eosinophilic cytoplasm. In one-third of cases, psammomatoid calcifications are seen. Round to oval nuclei and moderate membrane irregularity with vesicular to clear chromatin, similar to the nuclei seen with papillary thyroid carcinomas, are observed. Mitotic figures are uncommon and necrosis is rarely seen. No perineural and angiolymphatic invasion is noticed [1,4].

As for immunohistochemical staining, NPACs show epithelial membrane antigen (EMA), cytokeratin 5/6, and often cytokeratin 7 positive expression. S-100 protein expression is seen focally in many cases [1,2]. Cytokeratin 19 and TTF-1 may express in some neoplasms [1,6]. In these, NPAC is referred to as thyroid-like low-grade NPAC. Thyroid-like low-grade NPAC represents a small proportion of low-grade NPACs and it mimics metastatic papillary thyroid carcinoma [4,5,7]. Thyroglobulin negativity is seen in these malignant tumors [1,4,5].

Differential diagnosis includes the papillary type of intestinal-type adenocarcinoma, low-grade papillary adenocarcinoma of salivary gland origin, papillary thyroid carcinoma, and papilloma. Papillary type of intestinal-type adenocarcinoma is a primary sinonasal epithelial malignant tumor that histologically resembles intestinal adenocarcinoma. It is the second most common type of sinonasal adenocarcinoma following adenoid cystic carcinoma. The ethmoid sinus is the most common location (40%), followed by the nasal cavity (25%) and the maxillary antrum (20%) [8]. An irregular exophytic mass bulging into the nasal cavity or the paranasal sinuses, often with a necrotic and friable appearance, is revealed through nasal endoscopy in most cases. Male predominance has been recorded in the literature with a 4:1 ratio and the neoplasm tends to affect older patients (mean age of 60 years). A strong association is found with chronic exposure to wood [9,10] or leather dust [11]. Nasal obstruction, rhinorrhea, and bleeding are the most commonly reported symptoms. On histopathological examination, this neoplasm tends to be less glandular and more papillary. Columnar and goblet cells cover the papillae, the latter of which are sparse to absent in NPACs. The papillary type of intestinal-type adenocarcinoma may also contain Paneth cells [10,12].

Low-grade papillary adenocarcinoma of salivary gland origin predominately affects the minor salivary glands. It arises submucosally rather than from the surface epithelium. It may also occur in the major salivary glands in less than 5% of cases [13]. It is the second most common salivary gland carcinoma of the palate and oral cavity and it can rarely appear in the nasopharynx. Females are more frequently affected by the neoplasm (female to male ratio of approximately 2:1) and it occurs over a wide age range (reported ages: 19-90 years) [13,14]. S-100 protein expression is usually positive in low-grade papillary adenocarcinoma of salivary gland origin. Perineurial invasion is frequent, being seen in 60-75% of cases [13]. This neoplasm tends to be more aggressive with frequent local recurrence (27%) and nodal metastases (17%) [15].

Papillary thyroid carcinoma is the most common type of thyroid carcinoma. It can also occur in the thyroglossal duct, the lingual thyroid, or ectopic thyroid tissue [16,17]. Females are predominantly affected with a ratio of approximately 3:1 [18]. NPACs may easily be considered metastatic papillary thyroid carcinomas [4,5]. Thyroglobulin negativity in NPACs helps to distinguish them from papillary thyroid carcinomas. Additionally, TTF-1 may be positive in both neoplasms. Thus, TTF-1 might be useful in the differentiation between thyroid-like low-grade NPAC and low-grade NPAC, but it is not useful for distinguishing primary low-grade NPAC from metastatic papillary thyroid carcinoma [4,5]. Papilloma deriving from the surface epithelium or of minor salivary gland origin is also included in the differential diagnosis [15].

Surgical excision of the tumor is considered the gold standard treatment modality for NPAC [1, 4, 5]. In most cases, radical surgical procedures for complete excision are not required, as the disease tends to be limited at presentation. Surgery alone has been used in most patients, although some have also undergone radiation therapy [1, 4, 5]. There are reports of patients undergoing radiation therapy before primary surgery and of others after radical surgery with no recurrence [4]. Although tumor cells seem to react to radiation therapy, a limited number of such cases are reported in the literature. Concerns are raised by the fact that radiation therapy has potential side effects and increases the risk of the appearance of a second primary malignant tumor [19]. Quality of life after nasal surgery for malignant tumors is generally considered good and most people did not report significant limitations [20]. The prognosis of NPACs is very good as patients have not developed recurrences or metastases [1, 4].

## Conclusions

NPACs are extremely rare primary malignant tumors derived from the nasopharyngeal surface epithelium. Surgical excision is reported to be the most appropriate treatment method. Endoscopic resection is an effective modality with low morbidity. More studies are required to clarify the role of radiation therapy in treatment. The rarity of the neoplasm makes meticulous follow-up imperative. The presence of an exophytic mass with a papillary or polypoid appearance in the nasal cavity could be easily underestimated. Thus, a timely diagnosis and treatment require high clinical suspicion. As NPAC resembles other entities, caution is also needed in providing an accurate diagnosis.
